# Surgical options for distal radius fractures of type C in elderly patients over 65 years old: a comparison of external fixation with Kirschner wires and volar locking plate

**DOI:** 10.1186/s13018-023-04162-0

**Published:** 2023-09-09

**Authors:** He Zhang, Man Liu, Si-Yu Duan, Hai-Rui Liang, Rong-Da Xu, Zhen-Cun Cai

**Affiliations:** 1 Department of Orthopedics Surgery, Central Hospital Affiliated to Shenyang Medical College, 5 Nanqi West Road, Shenyang, 110075 Liaoning China; 2 School of Basic Medical Sciences, Shenyang Medical College, 146 Huanghe North Street, Shenyang, 110034 Liaoning China

**Keywords:** Distal radius fracture, External fixation, Volar locking plate, Surgical outcome

## Abstract

**Background:**

ue to the lack of consensus on the optimal surgical treatment for distal radius fractures (DRF) in elderly patients over 65 years old, the purpose of this study was to compare the efficacy of external fixation (EF) with Kirschner wires and volar locking plate (VLP) in the treatment of DRF through a retrospective cohort study. We hypothesized that there would be no significant difference in overall complications and functional recovery between the two methods.

**Methods:**

We retrospectively analyzed 62 patients over 65 years old who underwent surgical treatment for C-type DRF between 2019 and 2022. Based on the different treatment methods, they were divided into the EF group and the VLP group. General data, inpatient data, and postoperative complications during follow-up were recorded. The X-ray images before surgery, after surgery, and at the last follow-up were analyzed, and the results of wrist motion range, Gartland–Werley wrist joint score, and DASH score were evaluated 6 months after surgery for both groups.

**Result:**

Thirty patients underwent closed reduction and external fixation combined with Kirschner wire fixation, while 32 underwent open reduction and VLP fixation. The EF group had significantly shorter operation time, intraoperative blood loss, injury-to-surgery time, and hospital stay compared to the VLP group (all *p* < 0.001). At the last follow-up, the radiographic parameters (ulnar variance and radial inclination) and wrist joint function (wrist dorsiflexion and forearm supination) were better in the VLP group than in the EF group (*p* = 0.04, *p* = 0.01, *p* = 0.001, *p* = 0.02, respectively). However, there was no significant difference in overall Gartland-Werley wrist joint score, DASH score, and incidence of postoperative complications between the two groups (*p* = 0.31, *p* = 0.25, *p* = 0.47, respectively).

**Conclusion:**

For patients aged 65 and above with distal radius fractures (DRF) of type C, VLP and external fixation with Kirschner wires yield comparable functional outcome and complications rate at the short term. However, VLP allowed restoration of better radiological parameters.

## Background

Distal radius fracture (DRF) is a common type of fracture in adults, accounting for approximately 18% of all fracture types in the field of orthopedic trauma [1]. DRF is commonly caused by low-energy factors, and the risk of DRF is higher in elderly women, which is related to the higher incidence of osteoporosis in elderly women. With the aging of the population, it is expected that the incidence of DRF will further increase [2–4]. The purpose of DRF treatment is to restore wrist function as close as possible to the pre-fracture level. In clinical practice, closed reduction and immobilization with plaster or splint are the most common treatment methods for DRF [4]. Some studies have found no significant differences in the long-term prognosis between conservative and surgical treatment for DRF [5, 6]. However, some studies have also shown that more than 25% of patients receiving conservative treatment may experience secondary reduction loss, and the incidence of fracture malunion or nonunion is higher. Therefore, for adult intra-articular or unstable distal radius fractures, surgical treatment has better efficacy than conservative treatment [7, 8].

The main surgical treatment for DRF in adults is open reduction and internal fixation (ORIF) with a volar locking plate (VLP) being the most widely used due to its superior biomechanical properties [9]. External fixation (EF) is a traditional and commonly used treatment method for DRF due to its mature technique and ease of application, and many surgeons have applied it in the treatment of DRF [10]. However, there is still no consensus on the optimal surgical treatment for DRF [11, 12]. Although some related literature has compared the treatment of DRF with EF and VLP in the past, the conclusions drawn are not clear and even contradictory [10, 13–15]. This study aims to compare two surgical methods in elderly patients aged 65 and above with type C distal radius fractures through retrospective analysis of perioperative data, imaging data, and final wrist joint function, in order to guide clinical decision-making when selecting surgical treatment for this patient population. We hypothesize that there is no significant difference in overall postoperative complications and functional recovery between the two surgical methods for type C distal radius fractures in patients aged 65 and above.

## Methods

Retrospective analysis was performed on patients with distal radius fractures admitted to the Department of Orthopedics in the Affiliated Central Hospital of Shenyang Medical College from September 2019 to September 2022. All patients are provided with detailed information regarding the differences between conservative treatment and surgical treatment prior to admission, and they are given the freedom to choose their preferred treatment option based on their own wishes. Inclusion criteria were age of 65 years or older, fresh closed fractures, and X-ray showing C-type distal radius fractures. Exclusion criteria were pathological fractures, lost to follow-up, and inability to perform functional assessments due to other diseases. In this study, 72 patients met the inclusion and exclusion criteria, and 10 patients were lost to follow-up before 6 months postoperatively. A total of 62 patients were included in the final analysis, of which 30 patients treated with Kirschner wire combined with external fixation were defined as the EF group, and 32 patients treated with volar locking plate internal fixation were defined as the VLP group. General information of the two groups of patients was recorded, including age, gender, time from injury to surgery, length of hospital stay, and last follow-up time.

### Perioperative period

All patients underwent preoperative imaging studies including anteroposterior and lateral radiographs of the affected wrist, CT scans with three-dimensional reconstruction to determine the extent of fracture displacement, and were selected based on the AO/OTA classification system for distal radius fractures [16]. The choice of surgical approach was mainly based on the surgeon's preference. All patients were placed in a supine position and received either brachial plexus block or general anesthesia, and a tourniquet was used for bloodless field. The affected limb was positioned on a side table with abduction. All surgeries were performed by the same experienced surgical team and surgical time, X-ray exposure, and intraoperative blood loss were recorded.

### External fixation (EF)

**Fig. 1:**
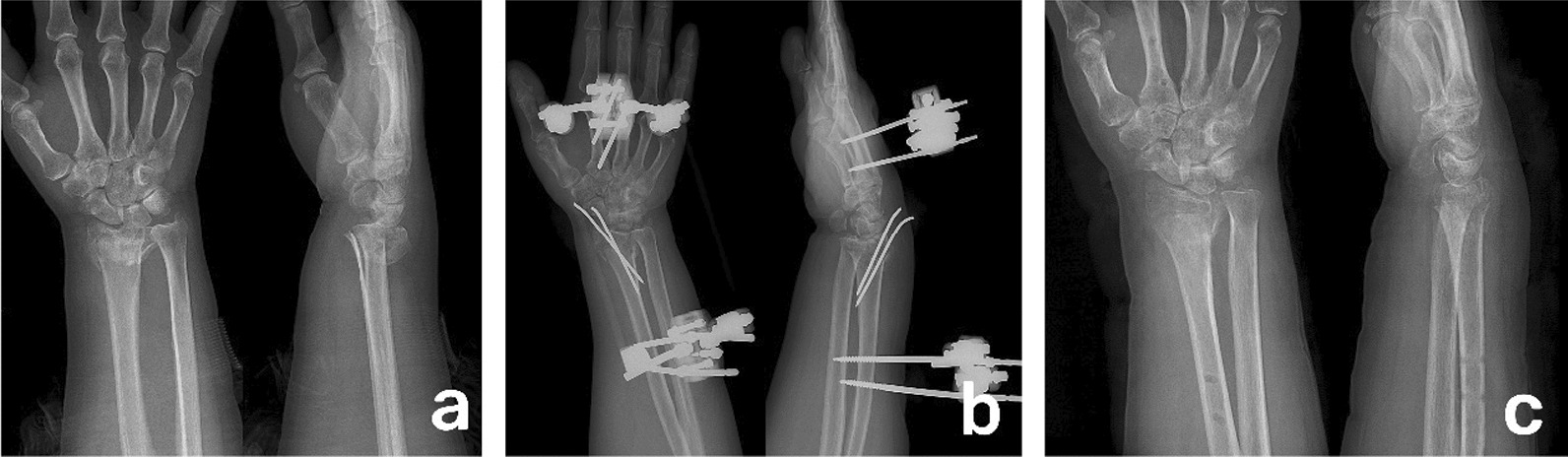
**a** Preoperative anteroposterior and lateral X-ray films of a patient with C3 type distal radius fracture. **b** Anteroposterior and lateral X-ray films on the first day after operation, showing adequate reduction and external fixation combined with Kirschner wire fixation of DRF. **c**. Anteroposterior and lateral X-ray films taken after 6 weeks, showing removal of external fixator and Kirschner wire

Two Schanz screws were drilled into the proximal radius, and two Schanz screws were drilled into the second metacarpal. Traction was applied to close and reduce the distal radius fracture, and the reduction was confirmed under fluoroscopy. One to two Kirschner wires with a diameter of 1.5 mm were implanted obliquely through the radial styloid process and into the cortical bone on the ulnar side to maintain fracture stability. The reduction of the distal radius fracture was reconfirmed under fluoroscopy. Then, an external fixation (Kangstaidi, China) device was used to fix the wrist joint in the desired position. Active and passive exercise of finger joint flexion and extension and thumb abduction was started immediately after surgery. The wound sutures and dressing were removed 2 weeks after surgery. The Kirschner wires and external fixation device were removed in the outpatient department at 6 weeks after surgery, and active and passive exercises of the wrist joint were started (Fig. 1a–c).

### Volar locking plate fixation

**Fig. 2:**
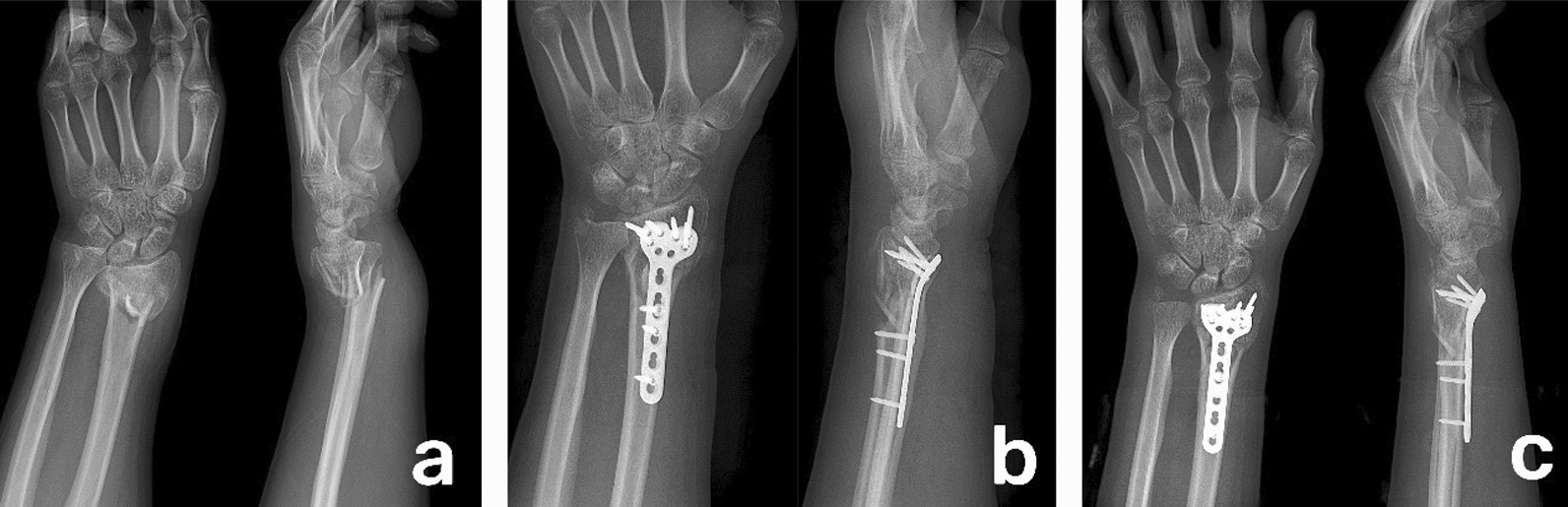
**a** Preoperative anterolateral X-ray of a patient with a C3 type comminuted fracture of the distal radius. **b** Postoperative first-day anterolateral X-ray with adequate reduction and fixation with a volar locking plate. **c** Anterolateral X-ray at 6 weeks postoperatively

The patient was approached with a longitudinal incision on the volar aspect of the affected wrist. The skin, subcutaneous tissue, and fascial layers were sequentially dissected, using a modified Henry approach, with attention paid to protecting the radial artery and median nerve. The pronator quadratus was released at its insertion, exposing the site of the distal radius fracture. Soft tissue within the fracture site was cleared, and the surgeon held the distal fragment with their fingers, while another surgeon held the proximal forearm. Traction was applied to reduce the fracture, and temporary fixation was achieved using a 2.0-mm Kirschner wire under fluoroscopic guidance to confirm the restoration of the radial inclination and volar tilt. The fracture was then fixed with a T-shaped locking reconstruction plate (Zhengtian, China) and screws, and the temporary Kirschner wire was removed. The pronator quadratus was repaired as much as possible. The affected limb was elevated on the first postoperative day, and early finger and wrist movement was started. Two weeks after surgery, the sutures and dressing were removed, and active and passive exercises of the wrist joint were strengthened (Fig. 2a–c).

### Postoperative follow-up

Postoperative follow-up was conducted at 2 weeks, 6 weeks, 3 months, 6 months, and 1 year after the surgery in outpatient clinics, with a minimum follow-up period of 6 months. Wrist joint anteroposterior and lateral X-rays were evaluated for patients on the first day after surgery and at the last follow-up. The imaging data were imported into a computer, and ImageJ software was used to measure and record the palmar tilt angle, radial inclination angle, ulnar variance, and radial height. At the 6-month follow-up, the range of motion of the wrist joint, including wrist flexion and extension and forearm pronation and supination angles, was measured with a goniometer for both groups of patients. The overall wrist joint function was evaluated using the DASH score system [17] and the Gartland–Werley wrist joint score standard [18]. The DASH score system is a questionnaire-based assessment of patients' daily activity ability, with a total score of 100 points, where 0 points indicate no disability, and higher scores indicate more severe disabilities. The Gartland–Werley score standard is based on a physician's evaluation of the patient, with a score range of 0 to 52 points, and higher scores indicate a poorer prognosis for wrist joint function. At each follow-up, based on the patient's self-description and the surgeon's examination, any complications that occurred in each patient were evaluated and recorded, including infection, nerve injury, chronic pain syndrome, fixation problems, tendinitis, hypertrophic scarring, traumatic arthritis of the wrist, etc.

## Statistical analysis

Continuous data were presented as mean ± standard deviation. Kolmogorov–Smirnov test showed that the samples did not follow a normal distribution, so we used the Mann–Whitney *U* test to compare the continuous variables between the EF group and the VLP group. Pearson chi-square test was used to compare categorical variables between the two groups. In these analyses, a *p*-value < 0.05 was considered statistically significant. All analyses were performed using SPSS26.0 software (IBM, Armonk, NY, USA).

## Results

### General information

A total of 62 patients were included in this study, with 30 patients (5 males, 25 females) receiving EF treatment with a mean age of 73 ± 6 years and 32 patients (8 males, 24 females) receiving VLP treatment with a mean age of 72 ± 7 years. There were no significant differences in gender and age between the two groups (*p* = 0.878 and *p* = 0.579, respectively). All patients had type C fractures, and there were no significant differences in fracture classification between the two groups (*p* = 0.579). Specifically, there were 7 patients with C1 fractures in the EF group and 10 in the VLP group, 12 patients with C2 fractures in the EF group and 14 in the VLP group, and 11 patients with C3 fractures in the EF group and 8 in the VLP group. All patients had a follow-up time of more than 6 months (Table 1).

**Table 1:** Comparison of general information of all patients

Grouping	Age (years)	Sex		Classification			Last follow-up time (months)
		Male	Female	C1	C2	C3	
EF	73 ± 6	5	25	7	12	11	7.0 ± 1.7
VLP	72 ± 7	8	24	10	14	8	6.8 ± 1.2
p	0.511	0.878		0.579			0.766

### In-hospital data

The time from injury to surgery, operation time, length of hospital stay, and intraoperative blood loss were all lower in the EF group than in the VLP group (all *p* < 0.001). The time from injury to surgery was 14.6 ± 6.8 h in the EF group and 65.9 ± 23.2 h in the VLP group. The operation time was 62 ± 10 min in the EF group and 79 ± 13 min in the VLP group. The length of hospital stay was 5 ± 2 days in the EF group and 7 ± 2 days in the VLP group. The intraoperative blood loss was 22 ± 9 ml in the EF group and 50 ± 24 ml in the VLP group. The number of intraoperative X-ray fluoroscopy was 9 ± 2 in both groups, with no significant difference (*p* = 0.875) (Table 2).

**Table 2:** In-hospital data of two groups

Grouping	Time from injury to surgery (hours)	Surgical time (minutes)	Length of hospital stay (days)	Surgical blood loss (ml)	X-ray fluoroscopy (frequency)
EF	14.6 ± 6.8	62 ± 10	5 ± 2	22 ± 9	9 ± 2
VLP	65.9 ± 23.2	79 ± 13	7 ± 2	50 ± 24	9 ± 2
p	< 0.001	< 0.001	< 0.001	< 0.001	0.875

### Imaging data

There were no significant differences in the palmar tilt angle, ulnar variance, radial height, and ulnar deviation on the first postoperative day between the two groups (*p* = 0.21, *p* = 0.46, *p* = 0.66, *p* = 0.53, respectively). At the last follow-up, there were no significant differences in the palmar tilt angle and radial height between the EF group and the VLP group (*p* = 0.22, *p* = 0.16, respectively). However, there was a significant difference in ulnar variance, with the EF group being 2.8 ± 2.9 mm and the VLP group being 0.1 ± 4.4 mm (*p* = 0.01), and in ulnar deviation, with the EF group being 19.9 ± 3.7° and the VLP group being 21.6 ± 3.6° (*p* = 0.04) (Table 3).

**Table 3:** Radiographic data of the two groups of patients

	Postoperative Day 1				Last follow-up			
Grouping	Palmar tilt angle (°)	Ulnar inclination angle (°)	Ulnar variance (mm)	Radius height (mm)	Palmar tilt angle (°)	Ulnar variance (°)	Ulnar variance (mm)	Radial height (mm)
EF	0.6 ± 6.5	21.2 ± 2.9	1.2 ± 2.3	11.1 ± 2.9	2.4 ± 5.5	19.9 ± 3.7	2.8 ± 2.9	10.6 ± 2.7
VLP	2.7 ± 7.2	21.4 ± 3.2	1.6 ± 2.1	11.6 ± 2.6	4.2 ± 6.2	21.6 ± 3.6	0.1 ± 4.4	11.6 ± 2.4
p	0.21	0.46	0.66	0.53	0.22	0.04	0.01	0.16

### Range of motion and scoring of wrist joint

At the 6-month follow-up after surgery, the wrist flexion function of the EF group was better than that of the VLP group, but the difference was not statistically significant (*p* = 0.41). The VLP group had better wrist extension and forearm pronation function than the EF group (*p* = 0.001, *p* = 0.02, respectively). The wrist supination function of the VLP group was also better than that of the EF group, but the difference was not statistically significant (*p* = 0.13). In the Gartland-Werley wrist score and DASH score, the EF group had higher scores than the VLP group, but the difference was not statistically significant (*p* = 0.31, *p* = 0.25, respectively) (Table 4).

**Table 4:** Range of motion and rating scales at 6 months postoperatively in both groups of patients

Grouping	Range of motion				Scoring scales	
	Flexion (°)	Extension (°)	Pronation (°)	External rotation (°)	Gartland–Werley (Score)	DASH (Score)
EF	65.3 ± 6.1	55.3 ± 9.2	78.6 ± 4.7	69.3 ± 7.2	4.1 ± 2.4	16 ± 12
VLP	63.9 ± 8.2	61.3 ± 5.5	81.2 ± 6.3	72.9 ± 7.9	3.4 ± 2.0	11 ± 7
p	0.41	0.001	0.02	0.13	0.31	0.25

### Complications

In the EF group, 9 patients had one or more complications, and in the VLP group, 7 patients had one or more complications. There was no significant difference in the overall incidence of complications between the two groups (*p* = 0.47). Four patients in the EF group and two patients in the VLP group had needle tract or wound infections, which were all cured by oral antibiotic treatment. One patient in the EF group and two patients in the VLP group experienced nerve injury, which all recovered spontaneously. One patient in the EF group developed chronic pain syndrome and received analgesic treatment. Three patients in the EF group experienced fixation problems that required re-adjustment, and one patient in the VLP group had a screw that penetrated the bone cortex due to excessive length. Two patients in the EF group and one patient in the VLP group had tendonitis, which was relieved by analgesics and rehabilitation treatment. Three patients in the VLP group developed hypertrophic scars. One patient in the EF group and one patient in the VLP group had traumatic arthritis of the wrist. No patients in the EF or VLP group experienced nonunion, and all fractures healed well (Table 5).

**Table 5:** Complications in both groups

Grouping	Overall complications	Specific complications						
		Infection	Nerve injury	Chronic pain syndrome	Fixation issues	Tendonitis	Hypertrophic scar	Traumatic arthritis
EF	9	4	1	1	3	2	0	1
VLP	7	2	2	0	1	1	3	1
p	0.47	0.35	0.59	0.3	0.27	0.52	0.09	0.96

## Discussion

The choice of treatment for distal radius fractures is influenced by multiple factors. In clinical practice, many patients are unwilling to tolerate the discomfort associated with long-term plaster casting and splinting, leading them to forgo conservative treatment with cast immobilization. This is especially true for patients with type C distal radius fractures who desire prompt pain relief and aim to reduce the risk of complications such as malunion, post-traumatic arthritis, and others. Consequently, both physicians and patients often opt for surgical treatment to expedite the healing process. In this study, our research group specifically compares the choice between two surgical methods for distal radius fractures in patients aged 65 and above with type C fractures. The choice of surgical approach for distal radius fractures has always been a matter of debate [11], particularly when comparing subtypes of distal radius fractures [12]. Through literature search, it was found that there is currently a lack of comparative studies between volar locking plate (VLP) and external fixation (EF) for type C fractures in patients aged 65 and above. In this study, our research group compared general characteristics, in-hospital data, radiographic parameters, wrist joint function at 6-month follow-up, and complications during treatment between the EF and VLP groups. We analyzed the advantages and disadvantages of these two surgical approaches for treating distal radius fractures. In this study, it was found that the VLP group had relatively better imaging data and wrist joint activity at the last follow-up. The VLP group demonstrated greater wrist extension and forearm pronation function compared to the EF group (extension: VLP 61.3 ± 5.5° vs EF 55.3 ± 9.2°, *p* = 0.001; pronation: VLP 81.2 ± 6.3° vs EF 78.6 ± 4.7°, *p* = 0.02). The VLP group also had slightly higher forearm supination function compared to the EF group (VLP 72.9 ± 7.9° vs EF 69.3 ± 7.2°), but the difference was not statistically significant (*p* = 0.13). Wrist flexion function was slightly lower in the VLP group compared to the EF group (VLP 63.9 ± 8.2° vs EF 65.3 ± 6.1°), but again, the difference was not statistically significant (*p* = 0.41). Although there were numerical differences in wrist joint range of motion, these differences did not result in significant perceptible differences in daily life activities, except in situations where specific wrist movements were required and compared to the uninjured side. Therefore, we conclude that the differences in range of motion between the two groups do not have practical clinical significance.

Moreover, there was no significant difference in DASH score, Gartland–Werley score, and overall complications between the two groups (*p* = 0.31, *p* = 0.25, *p* = 0.47, respectively). Hooper et al. [19] conducted a retrospective study of 184 patients with closed DRF and found that the use of EF fixation after 7 days of fracture would lead to greater surgical difficulty and poorer reduction quality, and VLP fixation was recommended for fractures over 7 days. In this study, the EF group underwent surgery mostly on the day of or the day after admission, while VLP group patients often chose to undergo surgery after the swelling of the wrist subsided to avoid complications such as non-healing of incisions and infections. Therefore, the time from injury to surgery and hospital stay in the EF group were significantly shorter (both *p* < 0.001), and patients fixed with EF could relieve wrist pain as soon as possible. External fixation has a history of nearly a 100 years in the treatment of DRF [20], with the advantages of easy operation and minimally invasive. Therefore, the EF group had significantly lower surgery time and intraoperative blood loss than the VLP group (both *p* < 0.001), and avoided the trouble of second surgery to remove the implant. Dağtaş et al. [10] compared the efficacy of EF and ORIF in the treatment of bilateral DRF and found that the wrist joint function in the EF group was better than that in the ORIF group during the first 2 months of treatment, making it a good choice for surgical treatment of bilateral DRF. Zhang et al. [15] compared the efficacy of EF and ORIF in the treatment of DRF through a retrospective cohort study, and the results showed that external fixation can provide faster functional recovery and better functional outcomes than ORIF within 2 years.

Quadlbauer et al. [21] applied volar locking plates (VLP) for the treatment of distal radius fractures (DRF) and evaluated the effects of immediate postoperative functional exercises and standard plaster fixation followed by 5 weeks of functional exercises on the recovery of wrist joint function after surgery. They found that immediate postoperative functional exercises significantly improved wrist joint range of motion and grip strength compared to plaster fixation, without observing an increased risk of reduction loss or other complications. In this study, the VLP group underwent immediate postoperative functional exercises, including fist clenching and appropriate wrist flexion and extension movements on the first day after surgery. The EF group only underwent exercises for finger flexion and extension and thumb abduction immediately after surgery. After 6 weeks of external fixation, fracture healing was assessed by X-ray, and after good fracture healing, the external fixation frame was removed for systematic functional exercises. In previous reports on elderly patients with DRF, especially those with osteoporosis, the use of external fixation had a poorer effect on maintaining radial length and a higher risk of pin loosening [22, 23]. However, no pin loosening complications occurred in any of the EF group patients in this study. During the study period, we found that EF patients had many inconveniences during external fixation, such as difficulty dressing, hygiene, and sleep, and were unable to perform systematic functional exercises on the first day after surgery, which hindered early postoperative recovery. In this study, compared with VLP, EF had the advantages of simpler surgical operation and earlier pain relief, but it still had disadvantages such as relatively poor postoperative imaging parameters and wrist joint range of motion.

Pin site infection is a common complication of external fixation, which may develop into deep tissue infection or even osteomyelitis in the long term [24, 25]. In this study, both groups of patients had good fracture healing and no related complications such as nonunion or osteomyelitis. The advantages of EF over VLP, such as less bleeding, shorter surgery time, and no incision, cannot be ignored in line with modern minimally invasive surgery [26]. At the same time, for patients with widely comminuted C2 and C3 DRF, the use of EF treatment has the advantages of shorter surgery time and equivalent functional outcomes [26, 27]. Therefore, our research group believes that in clinical practice, external fixation should be the preferred treatment for distal radius fractures in patients with poor cardiopulmonary function who cannot tolerate long surgeries, as well as for patients with severely comminuted C2 and C3 DRF.

There are several limitations to this study. Firstly, it is a retrospective and non-randomized study. Secondly, there was heterogeneity between the two groups. Thirdly, all patients were from a single center. Lastly, the surgical selection in this study was primarily based on the preference of the surgeons, which may have influenced the outcomes.

## Conclusion

For patients aged 65 and above with distal radius fractures (DRF) of type C, VLP and external fixation with Kirschner wires yield comparable functional outcome and complications rate at the short term. However, VLP allowed restoration of better radiological parameters.

## Data Availability

The datasets generated and/or analyzed during the current study are not publicly available due to limitations of ethical approval involving the patient data and anonymity but are available from the corresponding author on reasonable request.
